# Is PiSS Alpha-1 Antitrypsin Deficiency Associated with Disease?

**DOI:** 10.1155/2010/570679

**Published:** 2010-06-28

**Authors:** Dawn McGee, Laura Schwarz, Rebecca McClure, Lauren Peterka, Farshid Rouhani, Mark Brantly, Charlie Strange

**Affiliations:** ^1^Division of Pulmonary and Critical Care Medicine, Department of Medicine, Medical University of South Carolina, 96 Jonathan Lucas Street, 812 CSB, MSC 630, Charleston, SC 29425-6300, USA; ^2^Division of Pulmonary and Critical Care Medicine, College of Medicine, University of Florida, Gainesville, FL 32610-0225, USA

## Abstract

*Background*. Alpha-1 antitrypsin deficiency (AAT) is an inherited condition that predisposes to lung and/or liver disease. *Objective*. The current study examined the clinical features of the PiSS genotype. *Methods*. Nineteen study participants (PiSS) and 29 matched control participants (PiMM) were telephone interviewed using a standardized questionnaire. Demographic features, cigarette smoking, vocation, medication history, and clinical diagnoses were compared. Statistical analysis was performed. Finally, a comprehensive literature review was performed by two investigators. *Results*. 12/19 (63.2%) study participants reported the presence of lung and/or liver disease compared to 12/29 (41.4%) control participants. There trended toward having a higher frequency of medication allergies in the study population (42.11% versus 20.69%). *Conclusions*. The PiSS genotype was associated with a similar incidence of obstructive lung disease to controls. Selective bias intrinsic in testing for AAT deficiency and the rarity of the PiSS genotype will make future study of this association dependent on population-based tests.

## 1. Introduction

Alpha-1 antitrypsin (AAT) deficiency is an inherited condition that predisposes to lung and/or liver disease. The risk for lung disease is increased by environmental exposures, particularly cigarette smoking. SERPINA1,the gene that codes for AAT, is located at 14q32.1 [1]. Currently, there are approximately 100 allelic variants identified. The most common allele is the M allele which codes for protease inhibitor (Pi) M protein. As an autosomal codominant genetic condition, most individuals are PiMM. The most common severe deficiency allele is the Z allele which, in the homozygous state (PiZZ), is associated with AAT plasma levels that are 15% of normal. The S allele is associated with AAT plasma levels approximately 60% of normal in the homozygous state [2]. In the United States, the estimated phenotypic prevalences are 1 in 17 PiMS, 1 in 36 PiMZ, 1 in 1058 PiSS, 1 in 1124 Pi SZ, and 1 in 4775 PiZZ. The estimated US prevalence of the PiSS genotype from population studies [3] would generate a PiS allele frequency in the US population of approximately 3.1%.

While there exists a significant amount of data regarding the risks for symptoms associated with certain AAT genotypes (PiMS, PiMZ, PiSZ, PiZZ), there is very little information published regarding PiSS risks. Therefore, the objective of the current study was to examine the clinical features of individuals that have a PiSS genotype.

Essential to an accurate clinical description of individuals with any AAT polymorphism is a random selection of the population to be studied. This feature is difficult to achieve for rare genetic variants. This report will characterize the personal and family histories of individuals that are PiSS who have participated in a large family testing program using fingerstick polymerase chain reaction (PCR) genotypes for AAT characterization, the Alpha-1 Coded Testing (ACT) Study. In addition, a separate population who has enrolled in the Alpha-1 Foundation Research Registry will be reported. Finally, a review of populations of individuals that are PiSS from other studies is performed.

## 2. Materials and Methods

Participants were recruited through the ACT study and the Alpha-1 Foundation Research Registry. The IRB approved written informed consent for these studies allowed for contact regarding future questionnaires and research. ACT participants were drawn from a cohort of individuals usually tested because of a family member with AAT deficiency. Individuals identified through the Alpha-1 Foundation Registry were drawn from a group of 3090 registry participants with severe AAT deficiency or the carrier state. Since individuals with symptoms are more likely to be tested for AAT deficiency, the participants of the ACT Study and the Research Registry do not represent population-based cohorts. Therefore, to approximate the same biases in a control population, each individual with the PiSS genotype was randomly age, sex, race, and US state matched to an ACT participant with the PiMM genotype as a control.

Study participants were telephone interviewed by a certified genetic counselor, from November 2007 through February 2009, with a standardized questionnaire to identify demographic features, cigarette smoking history, medication history, past and present clinical diseases, and surgeries. A detailed pedigree was obtained, including race/ethnicity and a history of fetal demise or stillbirth. Social history including vocation and artistic experience was collected.

Statistics were performed by JMP (SAS Institute, Cary, NC). A two-tailed Student's *t*-test for continuous variables and Pearson's Chi-Squared test for categorical variables were used for comparative statistics when comparators had more than five observations. A *P*-value <.05 was accepted as significant. No correction for multiple comparisons was made.

The Hardy-Weinberg statistics were applied to the genotypes detected in the ACT study to calculate the S allele frequency in that study population. The total number of S alleles detected in the ACT study (812) was divided by the total number of alleles (15320) thus determining the S allele frequency (5.3%). The S allele frequency was squared and then multiplied by the total number of participants in the ACT study (7660) to determine the predicted number of individuals with the PiSS genotype (21).

A comprehensive literature review was conducted by two investigators (DM, CS). A recent meta-analysis of chronic obstructive pulmonary disease (COPD) risk associated with PiS alleles [4] was the starting point for our evaluation since the 119 articles reviewed in detail reported only seven articles that mentioned cases of the PiSS genotype. These seven articles were reviewed for any other clinical information in addition to COPD. In addition, testing studies that reported outcomes on more than 100 patients were reviewed from the dataset of de Serres [5] to ascertain if information was included on individuals that were PiSS. References of articles that discuss cases of PiSS were indexed and translations of articles in other languages from English were obtained. Tables of general population and disease specific testing were constructed.

## 3. Results

The ACT study and the Alpha-1 Foundation Research Registry enrollments by genotype as of January 1, 2008 are shown in Table 1. The ACT study identified 34 individuals with the PiSS genotype. Eight of these individuals chose to not continue in the longitudinal outcome study after receiving their results per study protocol [6], and contact information was not available. Sixteen of the remaining 26 individuals were interviewed. One of the 26 identified individuals died prior to being interviewed. The remaining nine individuals were not interviewed due to incorrect contact information (*N* = 6) or did not return three telephone calls (*N* = 3).

Five individuals in the Registry were identified with the PiSS genotype. One of these five individuals was also in the ACT study. Therefore, four additional individuals were identified for the current study. Three individuals were interviewed. The remaining person did not return the researcher's phone calls on three occasions but reported asthma in the Registry database. Therefore, 19 individuals comprise the current PiSS study cohort.

### 3.1. Participants of Current Study

Table 2 shows patient demographics for the cohort of the PiSS genotype and the control participants with the PiMM genotype. Eighteen participants were non-Hispanic Caucasian. One ACT study participant was Hispanic. There was no difference in smoking status, smoking duration, secondhand smoking incidence, or secondhand smoking duration between participants with the PiSS genotype and the PiMM genotype.  Table 3 lists physician-reported diagnoses for COPD, asthma, and hepatic steatosis (fatty liver) and self-reported jaundice, frequent pneumonia, and bronchitis. Lung disease symptoms, present in 11 of 19 (57.9%) participants from the cohort of the PiSS genotype, were not statistically higher than lung disease symptoms in 11 of 29 (37.9%) participants in the control group (*P* = .17). In addition, there was no correlation in univariant or multivariant analyzes between genotype, cigarette smoking intensity or duration, and the presence of lung disease. Jaundice was not a reason for ACT study or Registry enrollment in any individual with PiSS.

Participants were also asked about other diagnosed conditions.  Table 4 represents the conditions that were reported in at least two individuals from either the study or control populations. Anxiety and/or depression and/or bipolar disorder were reported in 47.4% of the participants with PiSS and 51.7% of the participants with PiMM (*P* = .77). A variety of conditions were reported with unusual disease states reported in both the study cohort and the matched controls. For example, fibromyalgia, hemolytic uremic disorder, and Hirschsprung's disease were reported in single instances in the control population. Down syndrome, endometriosis, and Sjögren's syndrome were also reported in single instances in the study population.

To address the possibility that the PiSS genotype in a fetus is associated with decreased viability, a detailed pedigree was obtained from each participant in the cohort of the PiSS genotype. This included information regarding miscarriages and stillbirths. The number of miscarriages and the number of stillbirths reported in each participant's biological parents were compared to the general population incidences for miscarriages and stillbirths. A detailed pedigree was not obtained on one participant who was removed from this analysis. 

A total number of 34 conceptions and 29 live births were reported in the 18 participants in this cohort. A total number of 79 conceptions and 68 live births were reported in the biological mother of the participants with the PiSS genotype. Thus, the incidences of fetal loss are 14.7% and 13.9%, respectively. 

**Figure 1:**
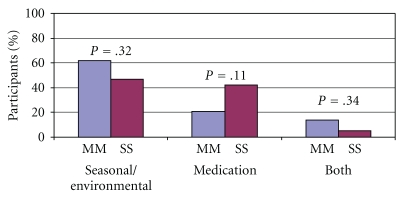
Percentage of seasonal/environmental allergies, medication allergies, or both seasonal/environmental and medication allergies in the study cohort (*N* = 19) compared to the controls (*N* = 29).

Participants were questioned regarding a history of allergies.  Figure 1 illustrates the percentage of participants in both the study and control populations that reported seasonal/environmental allergies, medication allergies, or a history of both seasonal/environmental and medication allergies. There were no differences in the reported allergy distribution although individuals with the PiSS genotype trended toward having more medication allergies (*P* = .11).

**Figure 2:**
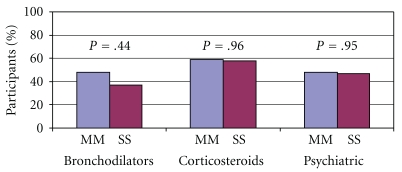
Lifetime medication use percentage in the study cohort (*N* = 19) compared to the controls (*N* = 29).

Figure 2 illustrates the percentage of participants in both the study and control populations that reported a lifetime history of using bronchodilators, corticosteroids, and psychiatric medications. There was no difference in use for bronchodilators (*P* = .44), corticosteroids (*P* = .96), or psychiatric medications (*P* = .95) from the control population.

The social history of each participant was also obtained, specifically the presence of a vocation that involved an artistic experience. The examples of artistic experiences listed in the scripted question included music, art, dance, theater, reading, or writing. Eleven (57.9%) participants with the PiSS genotype reported having an artistic experience compared with 17 (58.6%) participants with the PiMM genotype (*P* = .96).

### 3.2. Literature Review

A comprehensive meta-analysis of COPD risk associated with PiS alleles performed by Dahl et al. [4] reviewed seven studies with subjects identified as PiSS. Many research studies addressing the population genetics of Alpha-1 did not specifically list the number of individuals with the PiSS genotype found due to the small number of individuals detected. Table 1 lists research articles that did report the number of individuals with the PiSS genotype. 

The literature was also examined for studies that addressed the question of whether there is excess disease in a population of individuals with the PiSS genotype.  Table 5 lists four case-control studies examining the presence of COPD. Also listed in Table 5 are two case-control studies that reported the presence of allergy and nonatopic infantile asthma.

## 4. Conclusions

Few previous studies have characterized the clinical characteristics of individuals with the PiSS genotype, an AAT polymorphism that is associated with mean serum levels of AAT approximately 60% of predicted, similar to individuals with the PiMZ genotype. Defining the risk of COPD associated with the PiMZ genotype has been controversial [7, 17] despite having many more subjects (2%-3% of the population) to evaluate. The goal of this study was to define if there might be an independent risk for lung disease associated with the PiSS genotype and to evaluate whether additional clinical conditions not associated with lung or liver disease are increased in prevalence in individuals with the PiSS genotype. 

Individuals with the PiSS genotype are uncommonly encountered in clinical practice. However, the S allele frequency in ACT was 5.3%. When applying Hardy-Weinberg statistics to the S allele frequency of participants in the ACT study, we would predict 21 individuals with PiSS and found 34 individuals with PiSS. This suggests that there could be either an excess of symptoms that foster ACT study participation or family testing from families with S alleles. The PiSS genotype does not appear to be associated with excess fetal demise. The reported incidence of miscarriage and stillbirth in our cohort of individuals with the PiSS genotype is similar to the reported general population risk of miscarriage of 10%–15% [21]. Our population is not large enough and too biased to define whether a survival advantage exists for the PiSS genotype. 

Our study noted a higher than expected prevalence of lung disease in the individuals with the PiSS genotype; however, the same was noted in the control population. Eight of 16 individuals testing in the ACT study with PiSS had physician-diagnosed lung disease, with four of these asthma or COPD. Given the prevalence of both asthma and COPD, these cross-sectional data appear high. However, the incidence of asthma or COPD in the population of PiMM of the ACT study is 7/29 (24.1%). Assuming the null hypothesis that individuals with obstructive lung diseases are likely to test for AAT deficiency equally among genotypes, the slight excess in individuals with PiSS with COPD and asthma raises more questions than it answers. Unfortunately, the small sample sizes intrinsic to the PiSS polymorphism will make future studies of this association difficult to adequately enroll. 

This study also found a high prevalence of smoking in both the study and control groups with obstructive lung disease. We did our best to adequately evaluate smoking histories. As listed in Table 2, there was no statistical difference in the number of smokers in the PiSS cohort and the controls. Cigarette smoking is a retrospective data point and there could be differences in other environmental exposures that were not ascertained. Furthermore, cigarette smoking may not be a linear covariate in COPD. However, the small number of participants prevents a more comprehensive analysis of the interaction between genetics, environment, and the presence of lung symptoms.

There is incomplete characterization of whether liver disease is increased in individuals with the PiS allele. Liver disease has been reported in individuals with both the PiMS and PiSZ genotypes, although the frequency is less than in individuals with the PiZZ genotype [22]. Because cryptogenic cirrhosis is frequent in hepatology clinics, the association of these genotypes with clinical liver disease remains undermined. Our series would suggest that the incidence of jaundice (4 of 19 ACT study or Registry participants) is higher than predicted in a general population, although no cases of cirrhosis were identified. Although jaundice was not the reason for study enrollment in any individual, family members with AAT deficiency may have prompted testing and could have other genetic risks for liver disease.

Other associations of AAT alleles and clinical conditions have been rarely reported. Schemel recently suggested that the PiS allele was associated with a personality phenotype characterized by “intense creative energy” (ICE) [23]. ICE was created to describe an artistic personality, often associated with a mood disorder. A majority of individuals with at least one S or Z mutation in his cohort were artists of some persuasion. In fact, all three of the individuals in his cohort that were PiSS had an ICE phenotype [23]. For this reason, we asked the questions associated with this observation in an independent population finding. In our current study, 6/19 (31.6%) of individuals in the study cohort reported both a history of anxiety and/or depression and/or bipolar disorder and artistic experience. In our control population, the combination of artistic expression and mood disorder was 8/29 (27.6%). Furthermore, equal numbers of individuals in the study and control populations were receiving medications to treat psychiatric conditions. Thus we would suggest that there are not mood disturbances or an excess of psychiatric diseases in the PiSS genotype. 

There are many limitations to our study. Spirometry data were not available; therefore, the severity of lung disease was unable to be assessed. One possible reason for the high incidence of lung and/or liver symptoms could be due to sampling bias. The participants from the ACT study are more likely to be individuals with a family history of AAT deficiency or current clinical symptoms prompting AAT testing. The data for this study are derived from participant interviews and may be subject to miscommunication from their physicians. The individuals in the study and control groups differed in the years from diagnosis to time of interview. This discrepancy is a result of the fact that individuals chose when to participate in the ACT study and be tested for AAT deficiency. We also recognize that the number of individuals available for interview in this study is too low to fully characterize the PiSS genotype, since other clinical conditions might occur with low prevalence and reported conditions may not appear as significant in a larger sample size. Taking into account this small sample size further, we cannot make generalizations about the clinical significance of the PiSS genotype. However, this case study does provide an insightful perspective into the relatively unknown clinical picture of the PiSS genotype. 

The incidence of lung disease in the individuals with the PiSS and PiMM genotypes was higher than expected, however, not significantly significant. Therefore, larger epidemiological studies would be needed to determine if the PiSS genotype is associated with an increased risk for lung disease. In conclusion, we were not able to illustrate a difference in obstructive lung disease incidence between the PiSS genotype and the PiMM genotype in this largest case series reported to date.

## Figures and Tables

**Table 1:** Published research on Alpha-1 antitrypsin population genetics when PiSS was reported.

Author	*N*	PiMM	PiMS	PiSS	PiSS lung disease *N* (%)	PiSZ	PiMZ	PiZZ	Other	Unknown
General population studies										

Dahl et al. [7]*	9187	8182	500	12	0%	10	477	6	0	0
Horne et al. [8]	554	484	30	2	NA^†^	0	21	0	7	0
Gulsvik and Fagerhol [9]	1258	1102	60	2	100%	3	55	1	35	0

Referred population studies										

Brantly et al. [10]	442	194	59	16	NA	23	56	94	0	0
Webb et al. [11]	500	442	30	1	100%	0	18	0	6	3
Fagerhol and Hauge [12]	503	434	27	3	100%	2	14	3	20	0
Lochon et al. [13]	433	366	43	1	0%	1	18	1	3	0
Zorzetto et al. [14]	1399	900	297	8	NA	10	144	1	39	0

Current study										

McGee et al. Alpha-1 Coded Testing Study	7660	3954	549	34	8/16^‡^ (50.0%)	193	2632	238	60	N/A
Alpha-1 Foundation Research Registry	3090	0	26	5	5^§^ (100%)	144	777	1577	55	506

*Included in the MM genotype is the E polymorphism which is situated in the 3' noncoding enhancer binding region.

^†^NA: Not Available. This study did not indicate whether the individuals with the PiSS genotype were diagnosed with any lung/liver disease or had the presence of any symptoms.

^‡^Sixteen of 34 individuals tested were interviewed to obtain this frequency of any lung disease.

^§^Registry participants identified the presence of lung disease at study entry. Three of these are included in the study cohort of this paper. Lung diseases reported in all 5 individuals with PiSS include COPD, *N* = 2, Asthma, *N* = 3, and Frequent pneumonia, *N* = 2.

**Table 2:** Demographics.

	PiMM (*N* = 29)	PiSS (*N* = 19)	*P*-value
Age	46.4 ± 17.7	46.9 ± 15.5	.46
Race	28 White, 1 Native American	18 White	.76
Hispanic ethnicity	1 participant	1 participant	
Sex	21 Female, 8 Male	15 Female, 4 Male	.61
Smoking history	13	9	.95
% Lung disease	37.9%	57.9%	.17
% Liver disease	13.8%	26.3%	.28

One control participant reported both White and Native American races which makes 1 participant in each group not white.

**Table 3:** Lung and liver disease in 19 individuals with the PiSS genotype and 29 controls with the PiMM genotype.

	PiMM (*N* = 29)			PiSS (*N* = 19)		
Diagnosis	*N* (%)*	Age of diagnosis^†^	Age at interview^†^	*N* (%)*	Age of diagnosis^†^	Age at interview^†^

COPD^‡^	5 (17.24%)	46 ± 13.47	53 ± 13.78	3 (15.79%)	34.33 ± 6.03	62 ± 5.20
Asthma	4 (13.79%)	21.25 ± 14.5	41.25 ± 6.80	5 (26.32%)	24.2 ± 16.71	51.6 ± 14.91
Frequent Pneumonia	2 (6.90%)	8.5 ± 2.12	39.5 ± 10.60	3 (15.79%)	11.67 ± 9.71	48 ± 14.73
Frequent Bronchitis	1 (3.45%)	40	53	2 (10.53%)	15 ± 7.07	45 ± 8.49
Other lung disease	3 (10.34%)	NA	49.67 ± 30.17	4 (21.05%)	NA	50.5 ± 12.18
Jaundice	3 (10.34%)	0	29.33 ± 16.17	4^§^ (21.05%)	13.5 ± 15.93	64.5 ± 6.56
Hepatic Steatosis	1 (3.45%)	35	64	1 (5.26%)	34	35
None	17 (58.62%)	N/A	47.06 ± 17.61	7 (36.84%)	N/A	38 ± 16.39

*Participants may have >1 diagnosis.

^†^Mean ± standard deviation.

^‡^COPD diagnosis was physician diagnosed with either emphysema or chronic bronchitis.

^§^One participant had jaundice as a newborn and later diagnosed with Sjögren's syndrome at 48 years of age

and had elevated liver enzymes that resolved after a year of prednisone treatment.

N/A: not applicable, NA: Not available.

**Table 4:** Diagnoses with >2 individuals reporting disease.

Diagnosis*	MM *N* = 29	SS *N* = 19
Anxiety and/or depression and/or bipolar	15	9
Hypertension	7	5
Heart disease	3	3
Diabetes	2	3
Hypothyroidism	5	2
Irritable Bowel Syndrome	1	2
Osteoporosis	0	3
Arthritis	4	2
Hypercholesterolemia	2	2
Sleep problems	3	0
Acid Reflux/GERD	2	1
Migraines	2	0

*Diagnoses listed are ones reported by two or more participants.

**Table 5:** Case-control series of individuals with COPD and asthma in which at least one individual with the PiSS genotype has been identified.

	Case	Control
COPD		

Kueppers et al. [15]	0/114 (0%)	1/114 (0.9%)
Lieberman et al. [16]	3/965 (0.31%)	1/1380 (0.07%)
Sandford et al. [17]	2/193 (1.04%)	0/73 (0%)
Mittman et al. [18]	1/240 (0.42%)	0.1/240 (0.04%)

Allergy and nonatopic infantile asthma		

Ihrig et al. [19]	0/138 (0%)	5*/700 (0.71%)
Arnaud et al. [20]	2/298 (0.67%)	23/1653 (1.39%)

*Not indicated whether these five participants were PiSS or PiSZ genotypes.

## References

[1] *Protease inhibitor 1; pi*.

[2] ATS/ERS Statement (2003). Standards for the diagnosis and management of individuals with alpha-1 antitrypsin deficiency. *American Journal of Respiratory and Critical Care Medicine*.

[3] de Serres FJ, Blanco I, Fernández-Bustillo E (2003). Genetic epidemiology of *α*1 antitrypsin deficiency in North America and Australia/New Zealand: Australia, Canada, New Zealand and the United States of America. *Clinical Genetics*.

[4] Dahl M, Hersh CP, Ly NP, Berkey CS, Silverman EK, Nordestgaard BG (2005). The protease inhibitor PI*S allele and COPD: a meta-analysis. *European Respiratory Journal*.

[5] de Serres FJ (2002). Worldwide racial and ethnic distribution of *α*1-antitrypsin deficiency: summary of an analysis of published genetic epidemiologic surveys. *Chest*.

[6] Strange C, Dickson R, Carter C (2004). Genetic testing for *α*1-antitrypsin deficiency. *Genetics in Medicine*.

[7] Dahl M, Tybjærg-Hansen A, Lange P, Vestbo J, Nordestgaard BG (2002). Change in lung function and morbidity from chronic obstructive pulmonary disease in *α*1-antitrypsin MZ heterozygotes: a longitudinal study of the general population. *Annals of Internal Medicine*.

[8] Horne SL, Chen Y, Cockcroft DW, Dosman JA (1992). Risk factors for reduced pulmonary function in women; a possible relationship between Pi phenotype, number of children, and pulmonary function. *Chest*.

[9] Gulsvik A, Fagerhol MK (1979). *α*1-antitrypsin phenotypes and obstructive lung disease in the city of Oslo. *Scandinavian Journal of Respiratory Diseases*.

[10] Brantly ML, Wittes JT, Vogelmeier CF, Hubbard RC, Fells GA, Crystal RG (1991). Use of a highly purified *α*1-antitrypsin standard to establish ranges for the common normal and deficient *α*1-antitrypsin phenotypes. *Chest*.

[11] Webb DR, Hyde RW, Schwartz RH, Hall WJ, Condemi JJ, Townes PL (1973). Serum *α*1 antitrypsin variants. Prevalence and clinical spirometry. *American Review of Respiratory Disease*.

[12] Fagerhol MK, Hauge HE (1969). Serum Pi types in patients with pulmonary diseases. *Acta Allergologica*.

[13] Lochon B, Vercaigne D, Lochon C (1978). Pan-lobular emphysema: relationship with serum *α*1-antitrypsin levels, Pi phenotype and the HLA system. *Nouvelle Presse Medicale*.

[14] Zorzetto M, Russi E, Senn O (2008). SERPINA1 gene variants in individuals from the general population with reduced *α*1-antitrypsin concentrations. *Clinical Chemistry*.

[15] Kueppers F, Miller RD, Gordon H, Hepper NG, Offord K (1977). Familial prevalence of chronic obstructive pulmonary disease in a matched pair study. *American Journal of Medicine*.

[16] Lieberman J, Winter B, Sastre A (1986). *α*1-antitrypsin Pi-types in 965 COPD patients. *Chest*.

[17] Sandford AJ, Weir TD, Spinelli JJ, Paré PD (1999). Z and S mutations of the *α*1-antitrypsin gene and the risk of chronic obstructive pulmonary disease. *American Journal of Respiratory Cell and Molecular Biology*.

[18] Mittman C, Lieberman J, Rumsfeld J (1974). Prevalence of abnormal protease inhibitor phenotypes in patients with chronic obstructive lung disease. *American Review of Respiratory Disease*.

[19] Ihrig J, Schwartz HJ, Rynbrandt DJ, Kleinerman J (1975). Serum trypsin inhibitory capacity and Pi phenotypes. II. Prevalence of *α*1 antitrypsin deficiency in an allergy population. *American Journal of Clinical Pathology*.

[20] Arnaud P, Chapuis Cellier C, Souillet G (1976). High frequency of deficient Pi phenotypes of *α*1 antitrypsin in nonatopic infantile asthma. *Transactions of the Association of American Physicians*.

[21] American College of Obstetricians and Gynecologists (2002). ACOG practice bulletin. Management of recurrent pregnancy loss. Number 24, February 2001. (Replaces Technical Bulletin Number 212, September 1995). American College of Obstetricians and Gynecologists. *International Journal of Gynecology and Obstetrics*.

[22] Bowlus CL, Willner I, Zern MA (2005). Factors associated with advanced liver disease in adults with *α*1-antitrypsin deficiency. *Clinical Gastroenterology and Hepatology*.

[23] Schmechel DE (2007). Art, *α*1-antitrypsin polymorphisms and intense creative energy: blessing or curse?. *NeuroToxicology*.

